# Overall biomass yield on multiple nutrient sources

**DOI:** 10.1038/s41540-025-00497-y

**Published:** 2025-02-10

**Authors:** Ohad Golan, Olivia Gampp, Lina Eckert, Uwe Sauer

**Affiliations:** 1 Institute of Molecular Systems Biology, ETH Zürich, Zürich, Switzerland; 2 Life Science Zurich PhD Program on Systems Biology, Zurich, Switzerland

**Keywords:** Biochemical networks, Bioenergetics, Dynamical systems, Systems analysis, Bioenergetics

## Abstract

Microorganisms primarily utilize nutrients to generate biomass and replicate. When a single nutrient source is available, the produced biomass typically increases linearly with the initial amount of that nutrient. This linear trend can be accurately predicted by “black box models”, which conceptualize growth as a single chemical reaction, treating nutrients as substrates and biomass as a product. However, natural environments usually present multiple nutrient sources, prompting us to extend the black box framework to incorporate catabolism, anabolism, and biosynthesis of biomass precursors. This modification allows for the quantification of co-utilization effects among multiple nutrients on microbial biomass production. The extended model differentiates between different types of nutrients: non-degradable nutrients, which can only serve as a biomass precursor, and degradable nutrients, which can also be used as an energy source. We experimentally demonstrated using *Escherichia coli* that, in contrast to initial model predictions, different nutrients affect each other’s utilization in a mutually dependent manner; i.e., for some combinations, the produced biomass was no longer proportional to the initial amounts of nutrients present. To account for these mutual effects within a black box framework, we phenomenologically introduced an interaction between the metabolic processes involved in utilizing the nutrient sources. This phenomenological model qualitatively captures the experimental observations and, unexpectedly, predicts that the total produced biomass is influenced not only by the combination of nutrient sources but also by their relative initial amounts – a prediction we subsequently validated experimentally. Moreover, the model identifies which metabolic processes – catabolism, anabolism, or precursor biosynthesis—is affected in each specific nutrient combination, offering insights into microbial metabolic coordination.

## Introduction

Natural environments feature a broad spectrum of physicochemical parameters that collectively define constraints within which species survive and thrive. Of particular importance to microbial niche occupancy are the type of nutrients, their concentration, and temporal availability. For example, bacteria growing in a riverbed might experience continuous but low nutrient flux and high spatial homogeneity, while bacteria growing in pulsating environments, such as tidal wetlands or at the sea bottom, receive nutrients only sporadically at higher concentrations and high spatial heterogeneity^1,2^. Different physiological traits provide fitness advantages for different conditions. Homogeneous environments with high nutrient availability favor organisms with higher growth rates, allowing them to outcompete competitors for common resources or maximize utilization before nutrients are washed-out. In contrast, heterogeneous environments that are spatially structured or characterized by low nutrient concentrations favor organisms that utilize these nutrients more efficiently^3–6^. This dynamic explains why fast growing copiotrophs thrive when nutrients come in pulses, while slow growing oligotrophs excel in conditions where nutrients are steadily available at low concentrations.

The amount of biomass produced per consumed nutrient is physiologically defined as the biomass yield parameter, which describes the efficiency of nutrient utilization^7–9^. Theoretical models based on thermodynamic principles, known as ‘black box models’, predict the biomass yield for growth on a single nutrient source in homogeneous environments to high accuracy^10–12^. These models conceptualize growth as a single chemical reaction, considering nutrients as substrates and the produced biomass and secreted byproducts as products. By calculating the change in free energy of the overall reaction, these models can predict the biomass yield. Here, we adopt these models to qualitatively predict and subsequently test the overall biomass yield in batch cultures of *Escherichia coli*, where the outgoing flux of nutrients is limited, ensuring that all available nutrients are utilized, including reutilization of secreted byproducts. Given that microbes are frequently co-limited by multiple nutrients^13–15^, we investigate whether the overall biomass yield of a nutrient is influenced by the availability and metabolic properties of a second nutrient—specifically, whether it can be degraded for energy or used solely as a building block for biomass. Typically, black box models operate under the assumption that there are no mutual effects between nutrients; hence, the overall biomass yield of each nutrient is considered independent of the availability of others. We empirically tested this prediction experimentally by titrating a second nutrient into batch cultures grown on a single carbon source. Our results demonstrate that the overall biomass yield is not only dependent on the availability of the measured nutrient, but also significantly affected by the initial amounts of other nutrients, with the possibility of negative mutual effects. To explain these observations, we expanded the black box model to consider whether a second nutrient can be used for biomass synthesis or also degraded for energy generation—and included mutual effects between the metabolic processes associated with different nutrient sources. Our modified model qualitatively captures the experimental observations, providing insights into how nutrient combinations influence metabolism. Furthermore, using this model, we determined the mutual effects of various nutrient combinations on growth processes.

## Results

### Growth on a single nutrient source

**Fig. 1:**
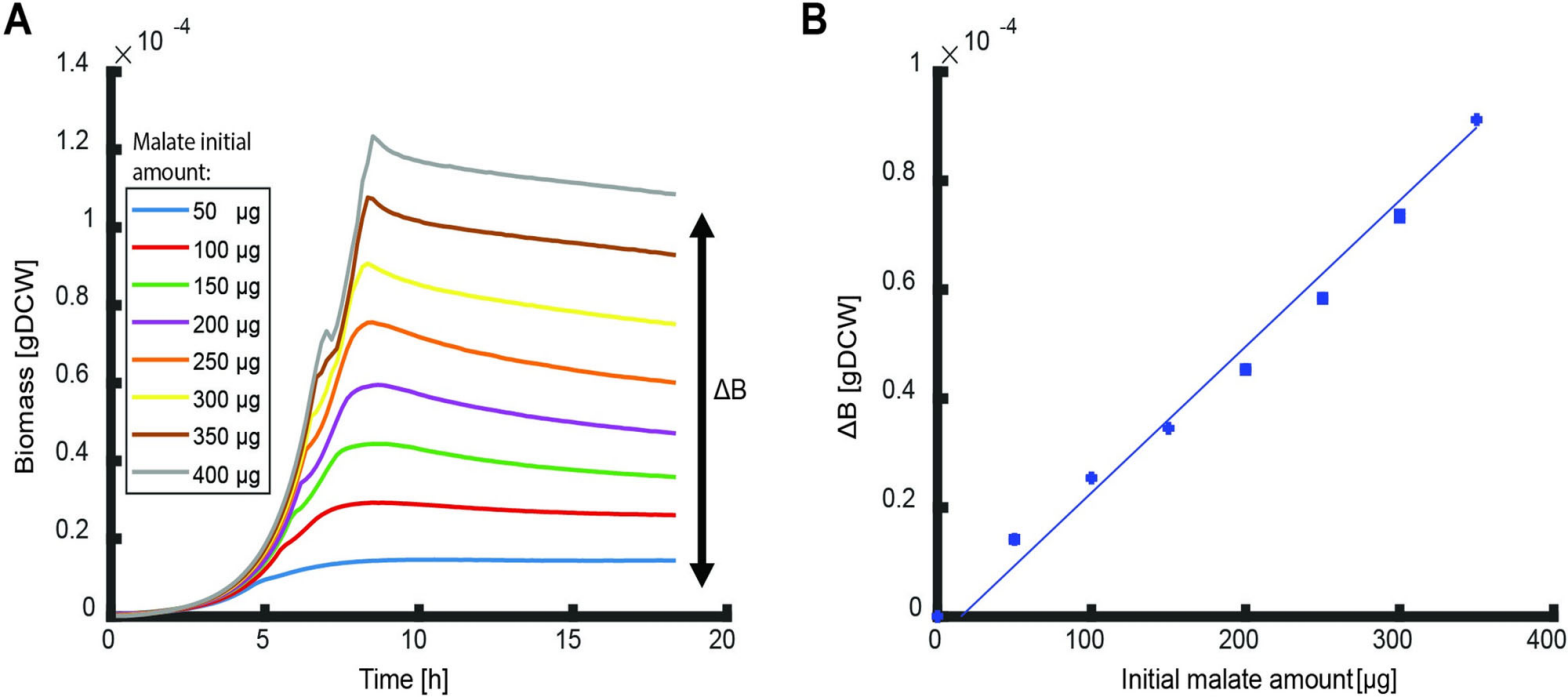
Overall biomass yield of malate. **A** Growth curves of *E. coli* for different initial amounts of malate. Curves are averages of three biological replicates. The produced biomass ($\Delta {BM}$) is the final biomass reached in stationary phase minus the initial biomass at inoculation. **B** The produced biomass of the different growth curves in (**A**) as function of the initial nutrient amount. The slope of the linear fit is the overall biomass yield (fit parameter R^2^ > 0.9). Bars of standard errors of the biological replicates are too small to be noticeable. Data for glucose and aspartate experiments are shown in Fig. S1.

The classical system to investigate the efficiency of nutrient utilization in environments where organisms have sufficient time to fully utilize all available nutrients are batch cultures. Here we follow growth of *E. coli* until depletion of the initial nutrient source and potential secreted byproducts when stationary phase is reached in M9 minimal medium with glucose, malate, or aspartate as sole carbon sources^16,17^. These carbon sources were chosen as respiro-fermentative, strictly respiratory, and a degradable biomass component. The produced biomass ($\Delta B$), that is the biomass reached at stationary phase minus the biomass at inoculation, was recorded as the optical density at 600 nm, converted to cellular dry weight using a predetermined conversion factor^18^, and plotted against the initial nutrient amount (Fig. 1; Fig. S1). The produced biomass shows a good linear fit to the initial amount of the sole carbon source (Fig. 1B) and as such, can be described by ref. ^16^:

1 $$\Delta B={Y}_{X/D}{N}_{D}$$

where ${N}_{D}$ is the initial amount of nutrient $D$ and ${Y}_{X/D}$ the overall biomass yield for organism $X$ on nutrient $D$ which describes the efficiency of full utilization of the available nutrient.

**Fig. 2:**
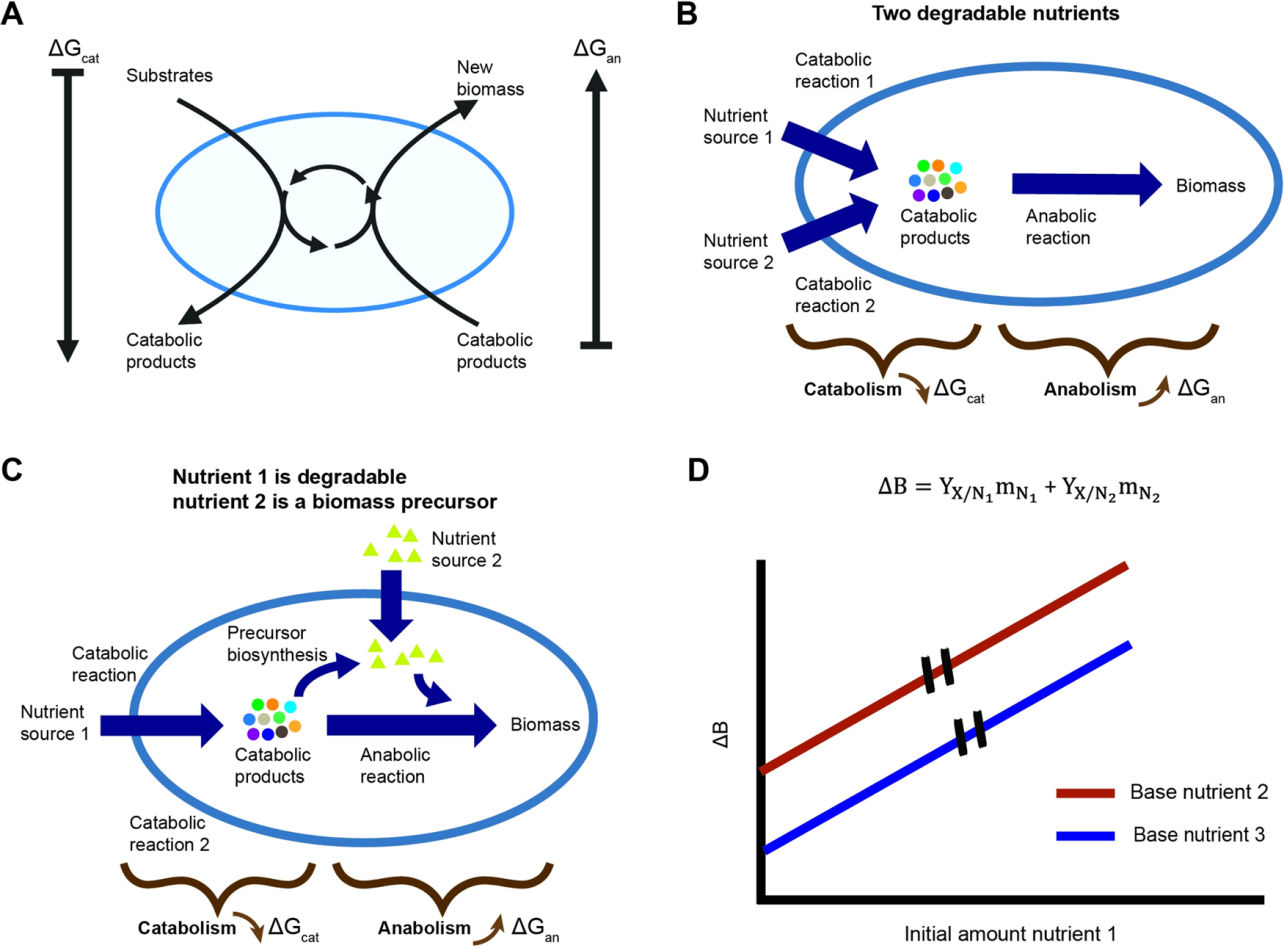
Expansion of black box model to include multiple nutrients. **A** The overall growth process is split into two reactions – a catabolic process in which free energy is released and an anabolic process in which new biomass is formed. The ring in the middle of the cell represents the coupling of anabolism and catabolism by ATP and other biochemical process. **B** Schematics of black box model expansion for two degradable nutrients. A catabolic reaction is added for each nutrient. **C** Schematics of model expansion for a combination of a degradable nutrient and a second nutrient that can only be used as a biomass precursor. The anabolic reaction is separated into two reactions – one for the biosynthesis of the biomass precursor and a second for the rest of the anabolic process excluding the biosynthesis reaction of the biomass precursor. **D** Black box model prediction for growth on two nutrient sources without mutual effect. The model predicts the produced biomass is a linear sum of the biomass gained from each nutrient. The overall biomass yield, the slope of the curve, is independent of availability of different nutrients.

To predict the produced biomass, we used a black box formalism^10^ that separates the growth reaction of chemotrophic organisms to a two-reaction process (Fig. 2A). The first is a catabolic reaction that releases Gibbs free energy by breakdown of nutrients. The second is the anabolic reaction that uses the released free energy for the synthesis of new biomass. The overall Gibbs energy dissipation $\Delta {G}_{X}$ of the growth process is given by (^10^, Fig. S1A text):

2 $$\Delta {G}_{X}=\frac{1}{{Y}_{X/D}}\Delta {G}_{{cat}}+\Delta {G}_{{an}}$$

where the subscripts *cat*, and *an* refer to the Gibbs energy of dissipation of the catabolic and anabolic reactions, respectively. Given that all secreted byproducts are utilized in the here investigated growth conditions, the free energy of the secreted byproducts can be set to 0, the overall biomass yield may be predicted as^10^:

3 $${Y}_{X/D}=\frac{\Delta {G}_{{cat}}}{\Delta {G}_{X}-\Delta {G}_{{an}}}$$

Combining Eqs. (1) and (3) predicts a linear correlation of the produced biomass as function of initial nutrient amount with a slope that depends only on the type of nutrient through$\,\Delta {G}_{{cat}}$. This prediction fits well with all measured nutrients and is consistent with previous results^16,17^ (Fig. 1B, Fig. S1).

### Growth on multiple nutrient sources

Since organisms typically encounter multiple nutrients in natural environments, we next asked whether the availability of one nutrient affects the overall biomass yield of another. To enable a black box model to capture such effects, we added another reaction that depends on the type of second nutrient: (A) degradable nutrients that first must be catabolized before they can be used, such as a sugar; (B) non-degradable nutrients that can be used only as a biomass precursor, such as the non-degradable amino acid methionine in *E. coli*; and (C) nutrients that can be both catabolized or used directly as a biomass precursor, such as the amino acid aspartate in *E. coli*. For the combination of two degradable nutrients the added reaction is catabolic (Fig. SA2 text, Fig. 2B). In this case, the overall Gibbs energy dissipation gives:

4 $$\Delta {G}_{X}=\frac{1}{{Y}_{X/{N}_{1}}}\Delta {G}_{{cat}}^{{N}_{1}}+\frac{1}{{Y}_{X/{N}_{2}}}\Delta {G}_{{cat}}^{{N}_{2}}\,+\Delta {G}_{{an}}$$

where $\Delta {G}_{{cat}}^{{N}_{i}}$ is the Gibbs energy of dissipation for the catabolic process of nutrient $i$. When the second nutrient source is a non-degradable biomass precursor, we split the anabolic reaction into two—a reaction for biosynthesis of the biomass precursor and a reaction for the general anabolic process (Fig. SA3 text, Fig. 2C). The overall Gibbs energy of dissipation in this case gives:

5 $$\Delta {G}_{X}=\frac{1}{{Y}_{X/{N}_{1}}}\Delta {G}_{{cat}}+\Delta {G}_{{an}}^{{bsyn}}+\Delta {G}_{{bsyn}}(1-{M}_{{utl}})$$

where $\Delta {G}_{{bsyn}}$ is the Gibbs energy of dissipation for synthesis of the biomass precursor and $\Delta {G}_{{an}}^{{bsyn}}$ is the dissipation energy for the general anabolic process minus that of the biomass precursor. The function ${M}_{{utl}}$ describes the ratio of available biomass precursor to that required to generate the produced biomass during the growth process. It is dependent on biomass precursor availability such that when all the necessary biomass precursor is available in the environment, the function assumes the maximal value of 1 and the cost for this precursor biosynthesis is alleviated.

Combining Eqs. (4) or (5) with Eq. (1) shows that regardless of the type of nutrient supplemented, the produced biomass is predicted to be a linear sum of the biomass gained from the available nutrients and the overall biomass yield of each nutrient is independent of the availability of others (Fig. SA2,3 text, Fig. 2D):

6 $$\Delta B={Y}_{X/N1}{N}_{1}+{Y}_{{X/N2}}{N}_{2}$$

where ${N}_{i}$ is the amount of nutrient $i$ in the growth medium and ${Y}_{X/{Ni}}$ is the overall biomass yield of nutrient $i$.

**Fig. 3:**
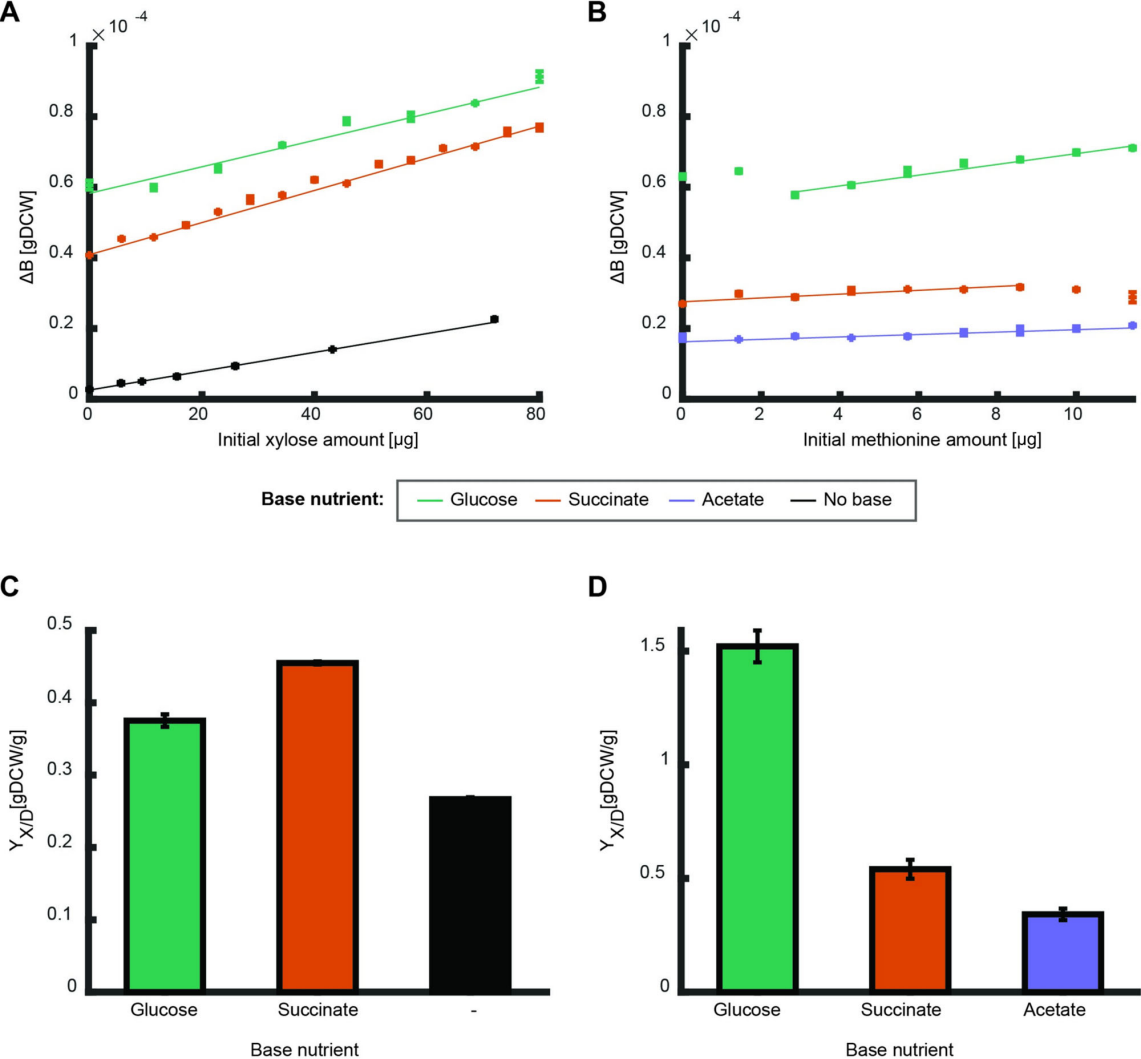
Produced biomass and biomass yield on different nutrient bases. **A, B** The produced biomass as function of initial amounts of the measured nutrients: xylose with or without different base nutrient sources (**A**, black– no base, green – 160 ${\rm{\mu }}{\rm{g}}$ glucose, orange –160 ${\rm{\mu }}{\rm{g}}$ succinate), and methionine with different base nutrient sources (**B**, green – 160 ${\rm{\mu }}{\rm{g}}$ glucose, orange – 80 ${\rm{\mu }}{\rm{g}}$ succinate, purple – 160 ${\rm{\mu }}{\rm{g}}$ acetate). Error bars depicting standard error for three biological replicates are in several cases not visible. Curves show a linear fit to the average of the three biological replicates of the linear region (fit parameter R^2^ > 0.9). **C**, **D** The overall biomass yield (slope of the fits above) for xylose and methionine for growth on the different base nutrients. Error bars depict error of fit parameter. The overall biomass yield is dependent on the nutrient base.

To test the prediction that the overall biomass yield of a nutrient is independent of the availability of others, we compared the overall biomass yield of *E. coli* for different nutrients, henceforth referred to as the measured nutrient, in the presence or absence of a second nutrient, termed the base nutrient. For this purpose, the initial amount of the measured nutrient was varied for each batch culture experiment between 0 and 1.2 g/l for xylose and 0 and 0.06 g/l for methionine, while maintaining constant initial amounts of the base nutrient: glucose, acetate, or succinate (Fig. 3). The produced biomass was plotted against the initial amount of the measured nutrient, and the overall biomass yield was determined as the slope of a linear fit of that curve (Fig. 3A, B). The initial amount of base nutrient determines the intercept with the Y-axis and was selected to ensure that the measured parameters remained within measurable range.

The overall biomass yield was highly dependent on the base nutrient. For xylose, the overall biomass yield was higher on succinate as base nutrient than on glucose or when used alone, and for methionine the overall yield was by far the highest on glucose (Fig. 3C, D). For most combinations, the influence of the second nutrient was monotonous across the tested concentrations, i.e., the overall biomass yield of the measured nutrient can be determined from the slope of a linear fit (Fig. 3A, B). An exception was the non-monotonous behavior of methionine as the measured nutrient in combination with glucose as a base nutrient (Fig. 3B). At low initial amounts of methionine (below 3 ${\rm{\mu }}{\rm{g}}$), increasing initial amounts of methionine unexpectedly decreased the produced biomass. In the higher range of initial amounts (above 3 ${\rm{\mu }}{\rm{g}}$), increasing methionine initial amounts increased the produced biomass linearly.

Thus, the overall biomass yield of a measured nutrient is dependent on the base nutrient, consequently black box theory cannot capture the produced biomass of multiple nutrient sources. To enable the model to describe such mutual effects, we expand it to include such effects phenomenologically. To do so, we coupled a function that is dependent on the combination of available nutrients to the Gibbs energy dissipation of each reaction in the growth processes. For simplicity, we assumed these functions are linear to the initial nutrient amount.

**Fig. 4:**
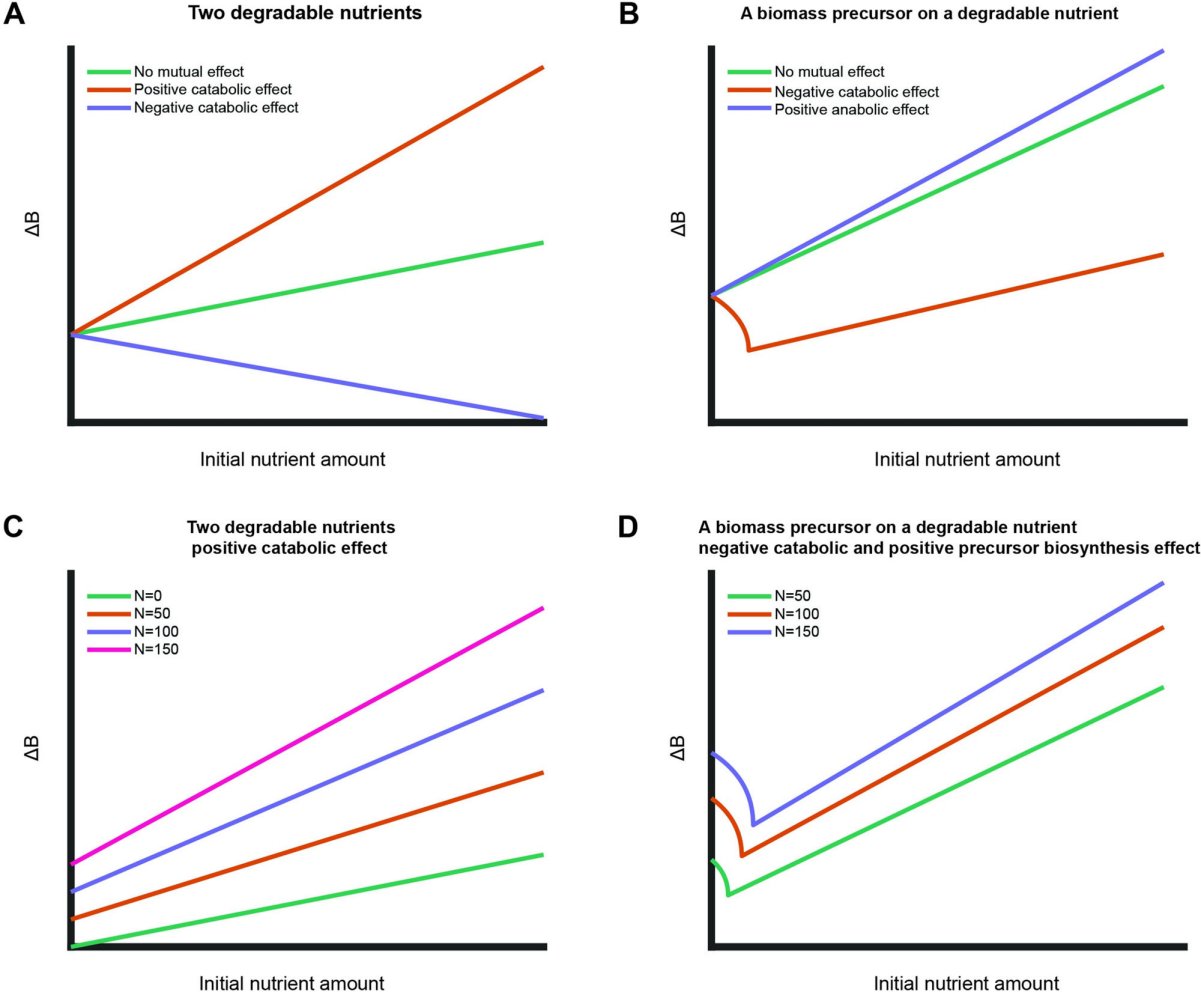
Expanded model solution space including mutual effects between two nutrients. **A, B** Simulations of expanded model with different mutual effects for growth on two degradable nutrient sources (**A**) and a biomass precursor in combination with a degradable nutrient (**B**). The initial nutrient amount of the base nutrient was kept constant in all simulations. **C** Simulations of expanded model for growth on two degradable nutrients for different initial amounts of base nutrient with positive catabolic mutual effect. The overall biomass yield (slope) increases with increasing initial amount of base nutrient. **D** Simulations of expanded model for growth on a biomass precursor and a degradable base nutrient with a negative catabolic effect and positive effect on precursor biosynthesis. Increased initial amount of base nutrient shifts the initial decreasing part and increases the slope (the overall biomass yield) of the linear part.

As such, the overall Gibbs energy dissipation of growth on two degradable nutrient sources is described as (Fig. SA4 text):

7 $$\Delta {G}_{X}=\frac{1}{{Y}_{X/{N}_{1}}}\Delta {G}_{{cat}}^{{N}_{1}}{f}_{{cat}1}\left({N}_{2}\right)+\frac{1}{{Y}_{X/{N}_{2}}}\Delta {G}_{{cat}}^{{N}_{2}}{f}_{{cat}2}\left({N}_{1}\right)+\Delta {G}_{{an}}{f}_{{an}}\left({N}_{1},{N}_{2}\right)$$

where ${f}_{{cat}}\left({N}_{i}\right)$, ${f}_{{an}}\left({N}_{i}\right)$ are linear functions to the initial amounts of nutrient source $i$, with coefficients${{\rm{m}}}_{{\rm{cat}}}^{{{\rm{N}}}_{{\rm{j}}}}\,,\,{{\rm{m}}}_{{\rm{an}}}^{{{\rm{N}}}_{{\rm{j}}}}$ respectively. These functions phenomenologically depict the mutual effect of the nutrient combination on the growth processes. Combining Eqs. (1) and (7) predicts the produced biomass:

8 $$\Delta B=\frac{\Delta {G}_{{cat}}^{{N}_{1}}{N}_{1}+\Delta {G}_{{cat}}^{{N}_{2}}{N}_{2}+\Delta {{\rm{m}}}_{{\rm{CAT}}}^{{{\rm{N}}}_{1}{N}_{2}}{N}_{1}{N}_{2}}{\Delta {G}_{X}-\Delta {G}_{{an}}{f}_{{an}}\left({N}_{1},\,{N}_{2}\right)}$$

where $\Delta {{\rm{m}}}_{{\rm{CAT}}}^{{{\rm{N}}}_{1}{N}_{2}}=\Delta {G}_{{cat}}^{{N}_{1}}{m}_{{cat}}^{{N}_{1}}+\Delta {G}_{{cat}}^{{N}_{2}}{m}_{{cat}}^{{N}_{2}}$ and ${f}_{{an}}\left({N}_{1},\,{N}_{2}\right)=1+{m}_{{an}}^{{N}_{1}}{N}_{1}+{m}_{{an}}^{{N}_{2}}{N}_{2}$. Given a mutual effect between nutrients, the produced biomass is thus made of three terms, two describing the direct effect of catabolism of the two nutrient sources and a third term describing the mutual catabolic effect depending on availability of both substrates. Although the last term of the model is based on the phenomenological observation, the range of model solutions is limited. Exploring this solution space shows that, depending on the type of mutualism, qualitatively different relationships are predicted between available nutrients and biomass formation (Fig. 4A) – a positive mutual catabolic effect increases the overall biomass yield (Fig. 4A, orange curve) while a negative catabolic effect decreases it (Fig. 4A, purple curve). The expanded model can capture the experimentally observed mutual effect of increased overall biomass yield with a positive mutual catabolic effect (compare increased slope for different base nutrients in Fig. 3A to the orange curve in Fig. 4A).

For growth on a combination of a degradable nutrient and a non-degradable biomass precursor, the overall Gibbs energy dissipation is described as (Fig. SA5 text):

9 $$\Delta {G}_{X}=\frac{1}{{Y}_{X/D}}\Delta {G}_{{cat}}{f}_{{cat}}\left(M\right)+\Delta {G}_{{an}}{f}_{{an}}\left(N,M\right)+\Delta {G}_{{bsyn}}^{M}\,{f}_{{bsyn}}\left(N\right)(1-{M}_{{utl}})$$

Where ${f}_{{cat}}\left({M}_{{utl}}\right)$, ${f}_{{an}}\left(N,{M}_{{utl}}\right)$, ${f}_{{bsyn}}\left({M}_{{utl}}\right)$ are linear functions with coefficients ${m}_{{cat}}$, ${m}_{{an}}^{N}$, ${m}_{{an}}^{M}$, ${m}_{{bsyn}}$ respectively. These functions depict the mutual effect between the nutrient sources on the Gibbs free energy of each growth reaction. Solving Eqs. (1) and (9) for the produced biomass gives a quadratic equation:

10 $$\begin{array}{l}\Delta {B}^{2}-\frac{\Delta B}{\Delta {G}_{A}}\,\left(N\Delta {G}_{{cat}}+{M}^{{\prime} }\left({m}_{{an}\,}^{M}\Delta {G}_{{an}}-\left(1+{m}_{{bsyn}}N\right)\Delta {G}_{{bsyn}\,}^{M}\right)\,\right)\\\qquad\,-\,N\frac{\Delta {G}_{{cat}}}{\Delta {G}_{A}}{m}_{{cat}}{M}^{{\prime} }=0\end{array}$$

Where $$\Delta {G}_{A}=\left(\Delta {G}_{X}-\Delta {G}_{{an}}(1-N{m}_{{an}\,}^{N})-\Delta {G}_{{bsyn}}^{M}\left(1+{m}_{{bsyn}}N\right)\right)$$ and $${M}^{{\prime} }={M}_{{utl}}\Delta B$$.

Unlike the solution for growth on two degradable nutrient sources, solving Eq. (10) for the produced biomass shows that a mutual effect between a biomass precursor and a degradable nutrient can give rise to non-monotonous solutions. Figure 4B explores the solution space of possible mutual effects between a precursor and a degradable nutrient. The case of a negative catabolic effect (Fig. 4B, orange curve) fits qualitatively well with the experimental observation of the biomass precursor methionine on glucose as base nutrient (Fig. 3B, green data points).

The coefficients of the linear functions depicting the mutual effect between the nutrients are a key output of the model since they infer how each combination of nutrients effects the different growth reactions. Fitting these coefficients to the experimental results of methionine growing with glucose as a base nutrient gives a qualitative fit to a negative value for the catabolic parameter (coefficient $${m}_{{cat}})$$, revealing that methionine decreases the catabolic efficiency of glucose. Furthermore, the overall biomass yield of methionine on glucose in the linear region is higher than that on succinate or acetate (Fig. 3D), suggesting a mutual effect on another metabolic process in one of these combinations, potentially the precursor biosynthesis processes (coefficient $${m}_{{bsyn}}$$). For all combinations of two degradable nutrients, the overall biomass yield increased as compared to growth on sole nutrient sources (Fig. 3C), a result that fits a positive mutual effect on the catabolic process (coefficient $${m}_{{cat}})$$.

An unexpected model prediction is noticeable in Eqs. (9) and (10) where the initial amounts of the two available nutrients are coupled in at least one term. Hence, the model predicts that the overall biomass yield of a measured nutrient depends not only on the availability of a base nutrient, but also on the relative initial amounts of the nutrients. For a combination of two degradable nutrient sources with a positive catabolic effect, as observed experimentally for xylose on the two base nutrients (Fig. 3A, C), the overall biomass yield is predicted to increase with increasing initial amounts of the base nutrient (Fig. 4C). For the combination of a degradable nutrient and a biomass precursor, such as methionine on glucose, with a negative catabolic effect and positive effect on precursor biosynthesis, the model predicts a shift of the curves for the non-linear part as well as an increase in the slope of the linear part with increasing initial amounts of base nutrient (Fig. 4D).

**Fig. 5:**
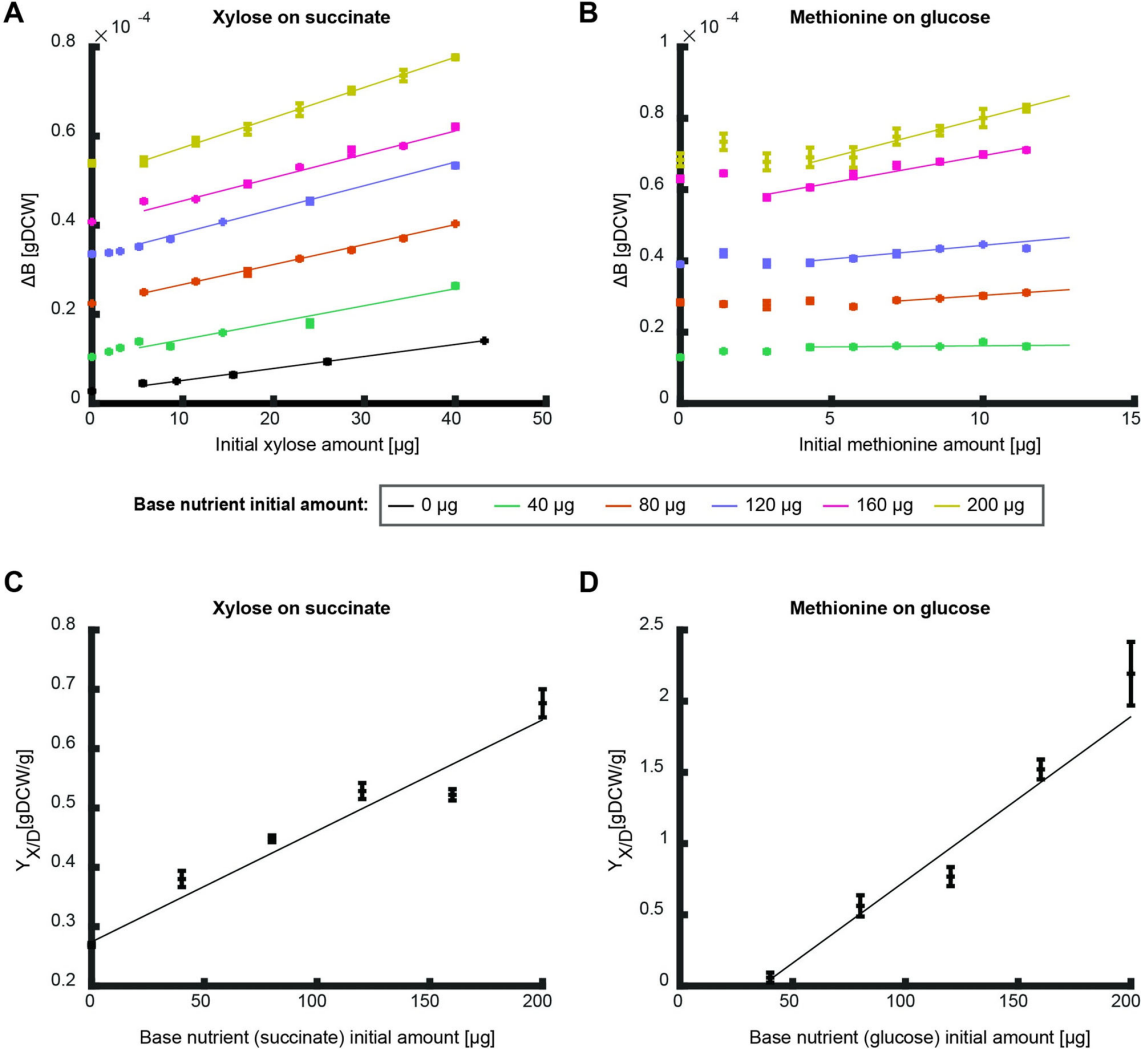
Effects of initial amounts of base nutrients on the overall biomass yield. **A, B** The produced biomass as a function of the initial amount of xylose (**A**) and methionine (**B**) at different initial amounts of the base nutrient succinate (**A**) and glucose (**B**). Data points are average of three biological replicates and error bars are standard error that are too small to notice. Curves are linear fits in the linear region to the average of the three biological replicates (fit parameter R^2^ > 0.9 for all fits except for methionine on 40 ${\rm{\mu }}{\rm{g}}$ glucose which showed a good fit to a constant (*p* < 0.05)). **C**, **D** The overall biomass yield as a function of initial nutrient amount as calculated from the conditions in (**A**, **B**). Curves show linear fit region (fit parameter R^2^ > 0.9). Error bars depict error of fit parameter.

To test these predictions, we determined the produced biomass on xylose and methionine as the measured nutrients on different initial amounts of succinate and glucose as the base nutrients, respectively (Fig. 5A, B). The overall biomass yield of xylose (i.e., slope of the curve) increased linearly with the initial amount of the base nutrient succinate (Fig. 5A, C). This observation fits well with the model prediction for a positive catabolic effect between two degradable nutrients (Fig. 4C). The curve of the produced biomass on methionine exhibits a more complex dependency on the initial amount of glucose as the base nutrient. Above $5\,{\rm{\mu }}{\rm{g}}$ methionine, the slope of all curves increased linearly with the amount of the base nutrient glucose, but below $5\,{\rm{\mu }}{\rm{g}}$ methionine there was no linear dependency, and the amount of base nutrient varied the curve shape (Fig. 5B, D). This observation fits well with the theoretical prediction (Fig. 4D) that this nutrient combination not only has a positive effect on the precursor biosynthesis reaction (i.e., the linear dependency at higher methionine supplementation), but also a negative catabolic effect where at low methionine concentrations, in some cases, more methionine leads to lower biomass gain.

Which mechanism underlies the negative catabolic effect of methionine on glucose? The growth curves followed the classical diauxic shift with exponential growth on glucose and a second phase on previously secreted acetate (Fig. S2A). For the example of 160 ${\rm{\mu }}{\rm{g}}$ glucose as the base nutrient (Fig. 5, pink curve), the first phase lasted 4–4.5 hours and growth on acetate resumed between 7 and 10 h (Fig. S2A). In both phases, the biomass gain (calculated as the biomass at the end minus the biomass at the beginning) increased linearly with methionine amounts greater than 2 ${\rm{\mu }}{\rm{g}}$ (Fig. S2B, C). The biomass gain was much higher than the trendline in the absence of or at very low methionine concentrations. During exponential growth on glucose in the first phase, methionine decreased the gain in biomass but increased the growth rate (Fig. S2D). Given the diauxic shift from growth on glucose to previously secreted acetate (Fig. S2E), the most plausible explanation for the higher biomass gains without or low methionine in the second phase is due to higher acetate secretion in the first phase. To test whether methionine supplementation indeed reduced acetate secretion, we varied acetate secretion rates by altering steady state growth through an inducible promoter for the glucose uptake gene *ptsG* that limits glucose uptake^19^. Comparing acetate secretion in the presence and absence of methionine shows that methionine indeed decreases acetate secretion (Fig. S2F). Thus, the negative catabolic effect of methionine on glucose catabolism appears to be a combination of a lower biomass gain during the first growth phase, with a higher growth rate and less acetate secretion, and a lower biomass gain in the second phase because less acetate was secreted.

At the lowest amounts of methionine (0 and 1.43 ${\rm{\mu }}{\rm{g}}$) we noted a shorter lag time for growth on acetate (Fig. S2A, compare red and black curves to the other curves). Growth with 1.43 ${\rm{\mu }}{\rm{g}}$ methionine was somewhat special as it followed the biomass trendline in the first growth phase but could not sustain the higher growth rate throughout this growth phase (Fig. S2A, red curve between 2 and 4 h), presumably because methionine was used up, which explains why its biomass gain in the second phase was indistinguishable from the no methionine condition (Fig. S2C). Consistently, 1.43 ${\rm{\mu }}{\rm{g}}$ methionine was below the amount necessary to produce the biomass reached at the end of the first growth phase (about 1.7 ${\rm{\mu }}{\rm{g}}$ of methionine is required to generate 0.8 ${\rm{gDCW}}$ of biomass^20^).

### Growth with a second nutrient that can be degraded and used as a biomass precursor

**Fig. 6:**
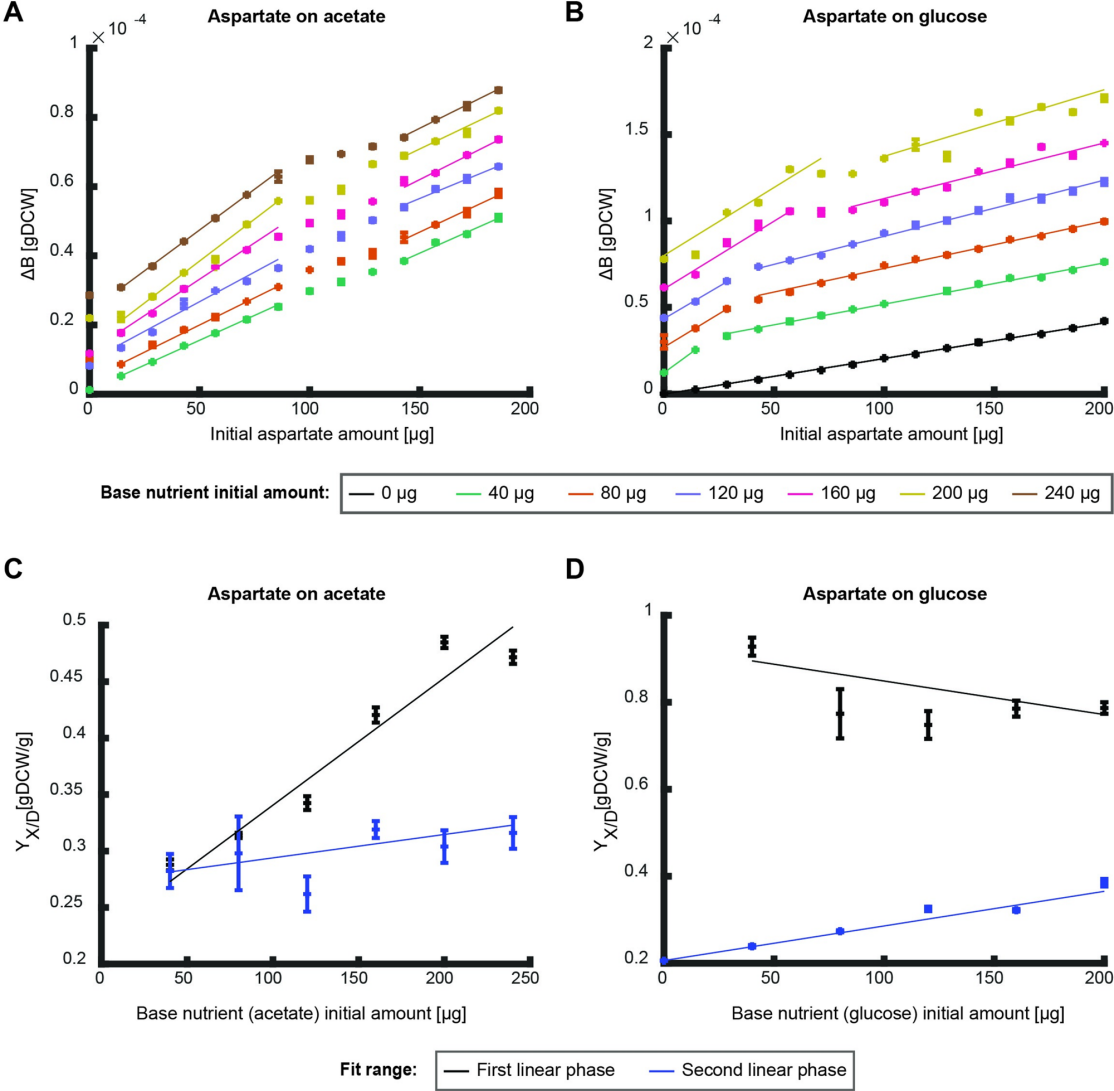
Effects of initial amounts of base nutrients on the overall biomass yield. **A, B** The produced biomass as function of initial nutrient amount of aspartate for different initial amounts of the base nutrient ((**A**) – glucose, (**B**) – acetate). Data points are average of three biological replicates and error bars are standard error that are too small to notice. Curves are linear fits to the average of the three replicates in the linear regions (fit parameter R^2^ > 0.9). **C**, **D** The overall biomass yield as function of initial nutrient amount as calculated from the conditions in (**A**, **B**) respectively. Black data points show the slope of the first linear phase and blue data points show the second. Curves show linear fit (fit parameter R^2^ > 0.9 for first linear fit on acetate and second linear phase on glucose, the other fits show a good fit to a constant (*p* < 0.05)). Error bars depict error of fit parameter.

So far, we focused on degradable nutrients or nutrients that can only be used as biomass precursors. Some nutrients such as degradable amino acids, however, can be directly used both as biomass precursors and energy source. Given the complex curves observed for the combination of biomass precursor and degradable nutrient, we expected that a degradable amino acid in combination with a degradable nutrient would also produce non-monotonous curves. To investigate the effects of such nutrient combinations, we measured the produced biomass on the degradable amino acid aspartate on different initial amounts of glucose and acetate as base nutrients (Fig. 6A, B). The combination of aspartate and acetate led to a complex curve with two linear phases separated by a double shift in slope at intermediate concentrations (between $100-150\,{\rm{\mu }}{\rm{g}}$, Fig. 6A). The first phase at low initial amounts of aspartate resulted in a linear slope that increases with initial amount of acetate while the slope of the second phase shows only a low dependency on acetate initial amounts (Fig. 6C). Aspartate on glucose also shows a complex curve with two linear phases (Fig. 6B). In this nutrient combination, the slope of the first phase is independent of the initial amount of glucose, yet the length of this phase increases with increasing initial glucose amounts (Fig. 6D). The slope of the second linear phase increases with increasing glucose initial amounts.

The complex behavior observed in these experiments cannot be captured with the mutual effect model presented here. We hypothesize that the overall biomass yield is influenced by the ratio of aspartate catabolized versus that utilized as a biomass precursor. The multiple utilization possibilities introduce an additional degree of freedom to the system, indicating that accurately modeling the behavior of these nutrients necessitates time-resolved intracellular flux information. Expanding the theoretical framework to accommodate these intricacies would require the introduction of additional assumptions, which may include assigning a preferential uptake sequence among the available nutrients, establishing a utilization preference for each nutrient, or implementing an optimization function.

## Discussion

In homogenous environments with low nutrient exchange, organisms fully utilize available nutrients and reabsorb previously secreted byproducts to sustain further growth, especially in the absence of competition. Here we asked whether the availability of one nutrient affects the utilization efficiency of others under such conditions. We showed that different nutrient combinations exert varying mutual effects on an organism’s ability to generate biomass, presumably by altering intracellular metabolism as well as secretion and reabsorption of secreted byproducts. These findings are relevant for understanding how nutrient co-limitation influences the efficiency of biomass formation in a given habitat^13^. While microbial utilization of multiple nutrients has been extensively studied – leading to significant findings such as the diauxic shift^21–23^ and the complex interplay of factors in multi-nutrient environments^24–26^ - these studies often fail to differentiate between different nutrient types and tend to focus on specific growth phases. Consequently, they may not adequately capture biomass gain across the entire growth curve.

Modeling microbial growth poses many challenges due to the inherent complexity of metabolic networks. These challenges include the accurate estimation of kinetic parameters, the integration of thermodynamic principles, and the management of computational complexity. Beyond stoichiometric models for steady state analyses, growth dynamics are typically investigated with kinetic models, which necessitates extensive knowledge of enzyme parameters—information that is typically not available or comes with considerable uncertainty^27–34^. This particularly complicates the modeling of complex growth curves, such as those observed when organisms utilize multiple nutrient sources, where nutrient concentrations fluctuate continuously, resulting in multiple growth phases. To address these challenges and achieve a more comprehensive understanding, we build upon previous black-box models based on fundamental thermodynamic principles. Rather than attempting to model the entire growth curve, our focus was on gaining insights from the starting and final biomass measurements, particularly the overall biomass yield. We expanded the black box model to account for the effects of different nutrient types and introduced a phenomenological representation of the mutual effects between nutrient sources. This expanded black box model qualitatively captured experimental observations and further predicts that the overall biomass yield of a nutrient is influenced not only by the availability of other nutrients but also by the initial ratio of the different nutrients.

Given the coarse granularity of a black box model, it does not identify the specific metabolic reactions. However, by fitting the model to experimental measurements, it can determine which coarse-grained metabolic processes – catabolism, anabolism or precursor biosynthesis—are responsible for the mutual effects observed in each nutrient combination. For instance, we found that in *E. coli* growing on glucose, methionine supplementation decreases the catabolic efficiency of glucose utilization and provide circumstantial evidence that the reduced efficiency may stem from a combination of methionine’s effect on the growth rate and reduced acetate secretion. The key strength of our black box model lies in its simplicity, requiring minimal input data while effectively capturing overall biomass yield and providing fundamental insights. However, it is limited by its exclusion of dynamic data, such as metabolic flux rates and growth rates during different growth phases. Further insights could possibly be gained by combining the thermodynamic black-box approach presented here with genome-scale metabolic models^27,35^ and detailed measurements of proteome allocation, alongside continuous metabolic flux estimates throughout the entire growth curve.

For all nutrient combinations, the initial amount of the base nutrient positively influenced the overall biomass yield of the measured nutrient, at least within some regions of the measured range. This finding aligns with previous reports indicating that supplementing growth media with casamino acids or yeast extract increases the carbon utilization efficiency of succinate or asparagine in batch culture experiments of *Enterobacter aerogenes* and *Pseudomonas perfectomarinus*^36^. Similarly, bacterial communities in aquatic systems were shown to more efficiently utilize mixtures of dissolved organic carbon sources, when compared to single-source utilization^37–41^. Moreover, it was shown that the carbon utilization efficiency of *Candida utilis*, *P. oxalaticus*, *Saccharomyces cerevisiae*, *Paracoccus denitrificans*, and *Thiobacilius versutus* exceeds theoretical predictions when a nutrient source that serves solely as an energy source is supplemented under balanced growth conditions^42–44^. It is tempting to conclude that the underlying reasons for this deviation from the theoretical prediction are similar across various experimental setups and measured parameters, such as biomass yield in a chemostat compared to overall biomass yield in batch cultures^7–9^. Our analysis suggests that the different nutrients influence each other’s catabolism, although the specific metabolic pathways involved remain unresolved. Several mechanistic hypotheses could explain the observed mutual nutrient effects, including the formation of storage compounds, growth-dependent proteome allocation, changes in maintenance energy requirements, regulatory feedback interactions between resources, and physiological trade-offs. These trade-offs may be related to limitations on nutrient uptake and utilization due to restricted membrane space or the need to allocate energy and resources specifically for nutrient acquisition. A comprehensive discussion of these potential mechanisms is provided in a recent review^13^.

Inherent to the black box models is the conception that biomass production is ultimately constrained by the available energy in the system. These models rely on a thermodynamic balance, where the energy derived from catabolic processes is compared to the energy expended in anabolic processes. While the primary focus of these models is the energy balance, the implications for carbon balance can also be inferred from the results. Given the conservation of carbon in the system, our findings suggest that nutrient combinations can significantly influence CO_2_ production during chemotrophic growth. Specifically, when a base nutrient enhances the yield of the measured nutrient, CO_2_ production per nutrient decreases. Conversely, if a base nutrient reduces the yield of the measured nutrient, CO_2_ production per nutrient utilized may increase. Understanding how nutrient combinations affect CO_2_ production raises broader ecological considerations^45,46^. Microbes metabolize a wide range of compounds, influencing the dynamics of organic matter utilization and CO_2_ emissions^47–49^, potentially impacting agricultural productivity, ocean nutrient balance, or global climate^49^. As most environments involve the utilization of multiple nutrients, investigating the interplay between different nutrients could facilitate more informed research in ecological systems. Furthermore, it may help mitigate the environmental impact of agriculture and the biotechnology industry by reducing CO_2_ emissions.

Nutrient utilization efficiency is important in various fields, including evolution, microbiome-host interactions, and synthetic biology. Different nutrient combinations can significantly influence the gut microbiome^50,51^. For instance, supplementation of certain nutrients, like amino acids, may sometimes lead to unexpected reduction of the overall biomass gain of gut bacteria^52^. Our findings demonstrate that amino acid supplementation affects the overall biomass yield, specifically highlighting the reduction caused by methionine. Although our analysis was conducted with a single organism, the abstracted processes are applicable to a variety of metabolic systems. This suggests that our methodology can be adapted to study growth of microbial consortia or even larger ecological systems. One practical application, could be in activated sludge systems, where a key challenge is to maintain low biomass yields^53^. Future research aimed at understanding the interactive effects between nutrients could enable our model to predict optimal nutrient combinations that minimize biomass yield in such systems. This would enhance the efficiency and sustainability of wastewater treatment processes. However, a crucial question remains: are there universal principles that govern these mutual effects, or is each nutrient combination influenced by a unique metabolic mechanism. Gaining insights into these phenomena could deepen our comprehension of ecological systems and foster improved interventions.

## Methods

### Strains and growth essays

In the growth essays the NCM3722 strain^54,55^ as used and in the acetate secretion essay NQ1243^19^. Each experiment was carried out in three steps: seed culture, pre-culture and experimental culture. For seed culture, one colony from fresh LB agar plate was inoculated into test tube with M9 minimal medium with 4 gr/l glucose and cultured in 37 °C shaking at 350 rpm for 8–9 h. The cell culture was then diluted to OD_600_ = 0.1–0.2 in pre-warmed shake flask with m9 minimal medium with the same base nutrient as the experiment and left to grow for two hours in 37 °C shaking at 350 rpm (pre-culture). The cell culture was then diluted to OD_600_ = 0.03–0.08 in pre-warmed 96 deep well plate with 1 ml. Each well contained medium with the experimental growth conditions (M9 minimal medium with nutrients according to experiment, each condition was set in triplicates) and mixed thoroughly. 200 µl cell culture from every well was then transferred to 96 deep well transparent essay plate and placed in Tecan microplate reader (Tecan infinite M200) for growth measurement. Microplate reader was programmed to maintain temperature at 37 °C, maximal shaking and measure OD_600_ every 10 min.

### Data analysis

The OD measured by the microplate reader was linearized using a premeasured calibration curve. Growth curves obtained in the microplate reader were compared to growth curves obtained in shake flask and were equivalent. The optical density was then converted to dry weight according to known calibration $0.396\frac{{gDW}}{{L\; OD}}\,$ ^18^. The final biomass point was recorded at 3-5 hours after maximal OD was reached. All linear fits were done to the average of the triplicate measurements by method of least-mean-square^56^ in the range that displayed a clear linear trend.

### Acetate secretion rate experiment

The experiment was done at 37 °C shaker shaking at 350 rpm in three steps: seed culture, pre-culture and experimental culture. For seed culture, one colony from fresh LB agar plate was inoculated into test tube with M9 minimal medium with 4 gr/l glucose and cultured in 37 °C shaking at 350 rpm for 8–9 h. The culture was then diluted in pre-warmed 96 deep well plate to an OD_600_ of 0.05–0.4 so that all cultures reached exponential phase at the same time. Each growth condition in the deep well plate was run in triplicates. All conditions contained m9 minimal medium, 4 gr/l glucose and different concentrations of the inducer for the glucose uptake promoter 3methyl-benzyl. Half of the growth conditions contained 0.1 gr/l methionine. Every 30 min, 40 µl culture from every well were collected and used to measure OD_600_ using Tecan microplate reader (Tecan Infinite M200). Another 100 µl culture from every well was collected, centrifuged at 15,000 rpm, the supernatant was collected and immediately frozen.

Supernatant were used to measure acetate concentrations using Acetate assay kit (Megazyme Acetic Acid Assay Kit). The slope of the plot of acetate concentrations versus OD_600_ for all replicates (multiplied with the measured growth rate) was used to determine the acetate secretion rate.

## Supplementary information

Supplementary information

## Data Availability

The experimental data of this study is available in Zenodo with the identifier 10.5281/zenodo.14398133.
